# Effects of chemical straighteners on the hair shaft and scalp^⋆^

**DOI:** 10.1016/j.abd.2021.02.010

**Published:** 2022-01-17

**Authors:** Joane Nathache Hatsbach de Paula, Flávia Machado Alves Basílio, Fabiane Andrade Mulinari-Brenner

**Affiliations:** Department of Dermatology, Complexo Hospital de Clínicas, Universidade Federal do Paraná, Curitiba, PR, Brazil

**Keywords:** Alopecia, Hair, Hair diseases, Hair preparations, Keratins, hair-specific

## Abstract

**Background:**

The effects of chemical straighteners on the scalp and hair shaft are not fully known, although such substances are widely used. Hair straightening became popular in Brazil with the use of formaldehyde and its derivatives, despite the prohibition by the current legislation.

**Objective:**

To identify changes in hair shaft and scalp caused by the use of chemical straighteners.

**Methods:**

A search was performed using keywords in three databases from 03/16/2020 to 05/20/2020, with publications between the years 2000 to 2020. After applying the inclusion and exclusion criteria, 33 articles were selected for review.

**Results:**

In some studies, hair relaxers were associated with eczema, desquamation, pain, burns, and inflammation in the scalp. Hair loss, damage to the shaft, alteration in the color of the hairs and in the composition of their amino acids were observed. Findings are variable across the studies.

**Study limitations:**

The search was restricted to three databases, in two languages, different study designs were accepted.

**Conclusions:**

Straightening techniques can have side effects, including scalp inflammation, damage to the shaft, and hair loss. Its long-term effects remain unknown and further studies are necessary.

## Introduction

The hair shaft consists of three layers with keratin in its composition: medulla, cortex and cuticle.^1^, ^2^, ^3^ Keratins are proteins rich in cysteine ​​residues.^1^, ^2^ Adjacent keratin chains join through tightly bonded disulfide bridges and other bonds such as Van der Waals, hydrogen bonds, and salt bonds.^1^

The hair shaft can be changed in shape by progressive or permanent chemical straightening. The first, in general, results from the use of acidic straighteners, have variable durability, and can present a cumulative effect with consecutive applications. The definitive ones, on the other hand, are permanent in the area of treatment and, as a rule, make use of alkaline substances.

Several compounds are used by the population to obtain chemical straightening, regardless of their approval by legislation. In Brazil, the technique called “progressive brushing” has spread with the use of acidic straighteners, such as formaldehyde and glutaraldehyde. The procedure has been disseminated worldwide as a Brazilian keratin treatment, among other names. Formaldehyde is classified as a colorless, highly toxic substance that, at room temperature, is a flammable gas.^4^ The terms methanal, methyl aldehyde, methylene oxide, formalin, oxymethylene, formic aldehyde, methylene glycol, paraformaldehyde, and oxymethane are listed as synonyms.^5^, ^6^ According to the National Health Surveillance Agency (ANVISA, *Agência Nacional de Vigilância Sanitária*), formaldehyde and glutaraldehyde are not listed as straighteners.^7^ Recently, substances that release formaldehyde only during the heating process have been used, such as methylene glycol, glyoxylic acid, timonacic acid.^5^, ^8^ The so-called “safe keratin treatments” use glyoxylic acid associated with carbocysteine ​​or amino acids, silicone derivatives and fatty acids.^9^

The ANVISA Normative Instruction -NI - number 64 of 07/27/2020 established, under the terms of the Resolution of the Collegiate Board (RDC, *Resolução de Diretoria Colegiada*) N. 409, its “List of allowed active principles in cosmetic products to straighten or curl hair”, which includes: thioglycolic acid and its salts, thioglycolic acid esters, sodium or potassium hydroxide, lithium hydroxide, calcium hydroxide associated with guanidine salt, sulfites, and inorganic bisulfites.^10^ These are the traditional hair straighteners, commonly referred to as relaxers. Although there are divergences, in general, this name is used to designate alkaline straighteners. They can be classified as lye (sodium hydroxide based), no-lye, ammonium thioglycolate, and bisulfites.^3^ Hydroxides are divided into lye and no-lye. Some authors categorize thioglycolate in the no-lye group.^11^

This article aims to identify the alterations in the hair shaft and scalp caused by the use of chemical straighteners.

## Methods

A search was carried out in the Pubmed, Lilacs and Scielo databases using the terms hair straightening, Brazilian hair treatment, Brazilian blowout, hair relaxer, formaldehyde hair, glyoxylic acid hair, thioglycolic hair, sodium hydroxide hair, calcium hydroxide hair, guanidine hair, potassium hydroxide hair, lithium hydroxide hair*, alisamento cabelo, escova progressiva, relaxamento cabelo, formaldeído cabelo, ácido glioxílico cabelo, tioglicolato cabelo, hidróxido de sódio cabelo, hidróxido de cálcio cabelo, guanidina cabelo, hidróxido de potássio cabelo, hidróxido de lítio cabelo*. Publications between January 2000 and May 2020 were included. The search period for the research was from 03/16/2020 to 05/20/2020. A total of 703 results were obtained.

The criteria for article inclusion was the topic of hair straighteners and their effects on the scalp and hair shaft. All results unrelated to hair straighteners were excluded by reading the article titles. Review articles, letters to the editor and doctoral theses were excluded after reading the abstracts of these articles, (but articles resulting from doctoral theses published in scientific journals were included). Articles on the term “permanent” were also excluded because, although the substances overlap with chemical straighteners, the technique consists of different steps. Articles related to occupational exposure to chemical substances in straighteners and publications that analyzed the formulations without investigating their effects on hair shafts or scalp were excluded.

Studies related to other hair techniques were not excluded when the information was mixed with that of straighteners (for example, the association of techniques) or when straightening was one of the studied subjects (in this case, the review only included information about straighteners). When the article jointly addressed the researched effects and systemic side effects, the latter were not detailed in the review.

**Figure 1:**
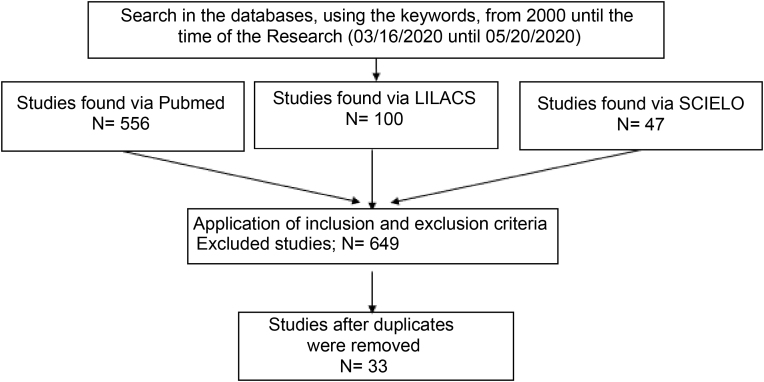
Article selection methodology.

Therefore, after applying the inclusion and exclusion criteria, 649 articles were excluded. The search resulted in 33 articles, after removing the duplicates, which were selected for reading and review. The methods are depicted in Fig. 1.

## Results

### Acid straighteners: formaldehyde, other aldehydes and formaldehyde derivatives

Formaldehyde can cause allergic contact dermatitis with progressive severity after each re-exposure, with the clinical presentation of eczema on the scalp and adjacent regions and swelling on the face.^12^

Cases of psoriasiform eczema on the scalp, face, neck, and upper trunk, occasionally with pustules, have been reported after treatment with a Brazilian keratin brushing. Dermoscopy revealed erythema and interfollicular and perifollicular desquamation, and histopathology showed psoriasiform and spongiotic patterns. The possibility has been raised that formaldehyde does not only causes lesions by allergic contact dermatitis or as a primary irritant, but it may induce reactions through other mechanisms.^13^

As for changes in the hair shaft, a comparative analysis of the mechanical properties of the hairs after treatment with formaldehyde and glyoxylic acid showed, in both, a reduction in hair breakage resistance, water uptake and retention, and mass. The hair treated with formaldehyde showed an increase in cuticle irregularity, enhanced by the use of heat. Glyoxylic acid showed even greater damage. These would be evidence of the harmful effects of the products on the hair shaft, despite findings of increased denaturation temperature, absence of increased oxidative damage, and presence of shine observed after treatments.^14^

Sanad et al. observed a better macroscopic appearance, correlated with the histopathological finding of reduced cuticle damage after the use of keratin straightener containing methylene glycol, in curly hair. On electron microscopy, two samples showed an increase in the density of intermacrofibrillar matrix in the cortex. Hair bleaching also occurred in some individuals, and hair loss was reported in 13.3%.^15^ In an evaluation of yak fibers and human hair by Boga et al., the sample treated with glyoxylic acid showed regular cuticles, whereas the sample treated with the basic solution and straightening iron showed more irregularities.^16^

Goshiyama et al., when studying formulas with glyoxyloyl-carbocysteine ​​and glyoxyloyl-amino acids of keratin, showed better straightening effect and more damage with more acidic solutions. Some of the side effects were changes in the strength, color, enthalpy and amount of tryptophan in the hairs. On electron microscopy, a film was observed on the treated hair, with a tighter cuticle, but which was partially removed after five washes.^17^ The results are shown in Table 1, Table 2.^12^, ^13^, ^14^, ^15^, ^16^, ^17^

**Table 1:** Studies on the effects of acid hair straighteners on the scalp.

Author	Study type	Population	Hair straightener	Scalp alterations
Van Lerberghe & Baeck.,^12^ 2014.	Case report	n = 1 (F:1, M:0)	Formaldehyde	Allergic contact dermatitis.
Gavazzoni-Dias et al.,^13^ 2016.	Case series	n = 7	Brazilian keratin treatment	Psoriasiform eczema.
				Biopsies (n = 4) compatible with psoriasiform dermatitis and psoriasiform spongiotic dermatitis.

n, Number of patients; F, Female; M, Male.

**Table 2:** Studies on the effects of acidic straighteners on the hair shaft.

Author	Study type	Population	Hair straightener	Hair shaft alteration
Leite & Maia Campos.,^14^ 2017.	Clinical trial	Curly hair samples	Glyoxylic acid and formaldehyde	Reduced resistance to hair breakage and diminished hair mass, less water uptake and retention, increased cuticle irregularity.
				Increased denaturation temperature, stable oxidative damage.
Sanad et al.,^15^ 2019.	Clinical trial	n = 30 (F:30 M:0)	Methylene glycol	Increased softness and shine at macroscopic examination.
				No changes in trichoptilosis or in cross-sectional fissures.
				Reduction of cuticle damage on histopathology, but no cuticle repair on electron microscopy.
				Increased density of intermacrofibrillar matrix in the cortex (n = 2).
				Shaft bleaching (n = 9).
				Hair loss (n = 4).
Boga et al.,^16^ 2014.	Clinical trial	Yak fibers and human hair	Glyoxylic acid	Persistence of cuticular regularity under electron microscopy.
Goshiyama et al.,^17^ 2020	Clinical trial	Dark brown curly hair samples	Glyoxyloyl-carbocysteine and glyoxyloyl -Amino keratin acids	Reduction in combing Work: 59.4% (pH 1.0), 33.3% (pH 2.0).
				Reduction of strand strength by: 15.7% (pH 1.0), 8.7% (pH 2.0). No statistical significance.
				Color changes and reduction in hair shaft enthalpy in both pHs.

n, Number of patients; F, Female; M, Male.

### Hair relaxers

The effects observed after more than one hair straightening with chemical relaxers include increased frizz, scalp desquamation, hair loss, thinning or weakening of the hair, hair dyschromia, trichoptilosis.^18^ In Kenya, 67% of women with Afro hair reported systemic and local effects after using different brands of hair relaxers. Among the dermatological manifestations, the following were mentioned: pain and/or desquamation on the scalp, allergic reactions, skin atrophy, weakening and/or change in hair color. The two most frequently mentioned effects were burns and hair loss. Despite the side effects, 55% chose to maintain some type of hair straightening method.^19^

The impacts of a Brazilian brand straightener, which had two products labeled as “chemical-free” and whose average tested pH was between 1.39 and 2.82, included serious effects such as hair breakage or loss in 95%, loss of more than 40% of hair in 56%, absence of repilation in 9%, in addition to other hair and scalp alterations.^20^ The pH of these products diverged from the alkaline standard of hair relaxers.^20^

In addition to the chronic effects on the scalp, secondary acute alterations have been reported, as in the case of a diabetic patient who, after using calcium hydroxide, developed a burning sensation on the skin with contact dermatitis, which progressed to infection with multiple abscesses.^21^ Also, patients who had acute irritative symptoms on the scalp during the relaxing process subsequently developed areas of cicatricial alopecia, especially in the vertex of the scalp, with histological findings suggestive of central centrifugal cicatricial alopecia (CCCA).^22^

Factors related to histopathologically confirmed CCCA were investigated by Shah & Alexis, and the use of hair relaxers was the most commonly reported element in patients with available data. However, due to the absence of a control group, it was not possible to establish an association.^23^ Gathers et al. and Olsen et al. found no relationship of relaxer use with the development of CCCA.^24^, ^25^ However, Olsen et al. found a statistically significant association between age at the first hair relaxing and the presence of alopecia when comparing the pattern without alopecia with the patterns of more advanced alopecia (probable CCCA). Hair loss does not prevent the use of relaxers, although it does decrease as the severity increases.^25^

Other types of alopecia are also a concern. In African-American girls, in which the prevalence of use of chemical relaxers was 42%, the previous use of relaxers doubled the risk of traction alopecia, and when associated with cornrow braids made 12 months before, the odds ratio exceeded five. Therefore, there is a risk in pulling relaxed hair into tight hairstyles.^26^ Some individuals may have structural changes related to major hair loss. In an atopic Nigerian woman, generalized proximal trichorrhexis nodosa was diagnosed after three episodes of universal hair loss related to relaxing procedures.^27^

Studies have explored the difference in the response of hair from different ethnicities to straightening. When evaluating cuticle damage after the process of shaking the hairs in water, in samples treated with thiol straightener, there was greater susceptibility to damage in the treated hair, proportionally higher in Chinese hair than in Caucasian hair. Cuticle damage was demonstrated on electron microscopy.^28^ Several procedures (including straightening), either associated or not, in natural black hair of different textures, did not result in a visible difference in clinical examination, on dermoscopy or optical microscopy. However on eléctron microscopy there was a significant difference between the groups, with more damage in those submitted to procedures compared to the control group, but not in relation to the type of hair or the specific treatment.^29^

The behavior of Caucasian and Afro hair submitted to straightening with thioglycolate diverged. The demand in Joules for the combing of Afro hair decreased, but there were no drastic differences in the other hair types. In the evaluation of color, there was a greater variation in dark brown and Afro-ethnic hair. Treatment-virgin hair showed greater tensile strength than straightened hair in all hair types, and the use of thioglycolate did not result in additional loss of protein or tryptophan, except in Afro hair. On electron microscopy, dark brown wavy, curly, and Afro hair showed mild opening of the hair scales, fissures, and breakage.^30^ Conversely, a study showed that Afro hair was more resistant to combined chemical stress. When straightening was combined with a commercial dye, the Afro cuticle and cortex were more resistant when compared to other ethnic groups. This was not expected, and perhaps hair mechanics themselves have been altered.^31^

Straightened hair is more vulnerable to other hair techniques added to straighteners. Areas of permanent alopecia were found where an artificial hair implant of Italian-knot type had been associated with hair straightening.^32^ The effects of the combination between hair dye and straighteners were studied in Caucasian dark brown and curly hair: the dye chosen by the researchers and straighteners increased, in relation to virgin hair, the loss of protein during washing. When the two were combined, there was no significant additional increase in these values, except for sodium hydroxide.^11^

Alterations were also demonstrated at the molecular level. Straight hair samples exposed to sodium hydroxide showed increased oxidative alterations, increased dehydroalanine formation, side-chain dehydration, and cysteine ​​damage, which likely corresponds to structural damage.^33^ Protein integrity, represented by the level of tryptophan and other degradation products, was studied in response to damage after straightening with lye relaxing cream. Fluorescence for tryptophan decreased, but there was no difference in kynurenines (one of the metabolites of tryptophan), which may mean that both tryptophan and its metabolites are chemically degraded.^34^

Afro and Caucasian white hairs were submitted to straightening with ammonia thioglycolate or triple treatment with the same straightener associated with discoloration with ammonia and hydrogen peroxide and also to the straightening iron use. After the treatments, important bonds were changed, with small differences between ethnicities. Straightening showed greater action on the band belonging to cysteic acid and resulted in lesions to the hairs, with cuticle irregularities and detachment and small undulations along them. The triple treatment showed hair shafts with irregular contours, cuticle detachment and deformation, as well as possible cortical damage.^35^

When comparing product A (lye) with product B (no-lye, with guanidine hydroxide), Mamabolo et al. showed a decrease in cystine in the hairs after treatment with both products, with a greater loss with the use of product B. The reduction in cystine was accompanied by greater straightening activity.^36^ There was no significant difference in the tolerability of the two products on the scalp.^36^

Khumalo et al. found a difference in the amount of four amino acids (cystine, citrulline, arginine and glutamine) between the groups with and without straightening. Cystine levels in treated hair were similar to those found in trichothiodystrophy.^37^

Focusing on inflammatory changes, Beach et al. collected some cytokines from the sebum of the scalp of women who underwent procedures with relaxers or remained with their natural hair. At sufficient levels for interpretation, IL-1α (pro-inflammatory) and IL-1ra (anti-inflammatory) were detected, and although cytokine differences between the vertex and crown may reflect a distinct predisposition of scalp sites to inflammation, it was not possible to demonstrate changes in specific inflammatory cytokines after use of relaxers.^38^ Tackey et al., using a three-dimensional epidermis model, compared lye and no-lye straighteners paired with untreated controls in relation to cytokines involved in the modulation of irritation after hair straightening. IL-1α reached higher levels at 24 and 48 hours after the use of a no-lye straightener. Both straighteners resulted in higher prostaglandin E2 (PGE2) levels than controls at all assessed periods, and the cytokine could account for sensory differences between different types of products.^39^

Conditioning and protective agents can minimize damage caused by straighteners, and their interference with straightening effectiveness is controversial.^40^, ^41^, ^42^ Findings are summarized in Table 3, Table 4, Table 5, Table 6, Table 7.^11^, ^18^, ^19^, ^20^, ^21^, ^22^, ^23^, ^24^, ^25^, ^26^, ^27^, ^28^, ^29^, ^30^, ^31^, ^32^, ^33^, ^34^, ^35^, ^36^, ^37^, ^38^, ^39^, ^40^, ^41^, ^42^, ^43^

**Table 3:** Studies that evaluated signs and symptoms from hair straighteners.

Author	Study type	Population	Hair straightener agent	Alterations in hairs and scalp
Shetty et al.,^18^ 2013.	Cross-sectional (questionnaire)	n = 90 (F:90, M:0)	Chemical relaxers	Increased frizz (67% of cases), thinning or weakening of the hair (40%), dyschromia (greying) of the hair (22%), trichoptilosis (17%).
				Scalp desquamation (61%) and hair loss (47%).
Etemesi BA.,^19^ 2007.	Cross-sectional (questionnaire)	242 (F:242, M:0)	Chemical relaxers	Weakening and/or changing of hair color.
				Scalp pain and/or desquamation, allergic reactions, thinning of the skin, burns and hair loss.
Kaur et al.,^21^ 2002.	Case report	n = 1 (F:1)	Calcium hydroxide	Contact eczema and secondary staphylococcal infection.

n, Number of patients; F, Female; M, Male.

**Table 4:** Study that evaluated signs and symptoms of a hair straightener (non-traditional acid straightener).

Author	Study type	Population	Hair straightener agent	Alterations in hairs and scalp
Swee et al.,^20^ 2000.	Cross-sectional (questionnaire)	n = 464 (F:457, M:5, non- identified: 2)	Acid straightener (non-traditional) with metallic salts	Hair breakage or loss (95% of cases), dry or coarse hair (70%), hair discoloration (28%).
				Dry scalp (53%), burning pain in the scalp (25%).

n, Number of patients; F, Female; M, Male.

**Table 5:** Studies that evaluated the association of hair straighteners with alopecia.

Author	Study type	Population	Hair straightener	Scalp alterations
Khumalo et al.,^22^ 2007.	Case series	n = 5 (F:5, M:0)	Sodium hydroxide (n = 3) and Guanidine hydroxide (n = 2)	Irritative symptoms and development of areas of cicatricial alopecia. Histological findings of perifollicular lymphocytic infiltrate, follicular fibrosis, and premature desquamation of the inner root sheath.
Shah & Alexis,^23^ 2010.	Case series	n = 69 (F:67, M:2) (64: data on hair care)	Chemical relaxers	Straighteners were the most common traumatic practice in patients with CCCA. It was not possible to establish an association due to the absence of a control group.
Gathers et al.,^24^ 2009.	Case-control study	n = 101 (F:101, M:0)	Chemical relaxers	There is no relationship between the use of relaxers, their frequency of application or complications from their use with the development of CCCA.
		CCCA Group (n = 51).		
		Group without cicatricial alopecia (n = 50).		
Olsen et al.,^25^ 2011	Case-control study	n = 529 (F:529, M:0)	Chemical relaxers	No association was observed between the use of straighteners or reaction to their use with extensive central hair loss.
				Association between age at first hair straightening and hair loss pattern when comparing pattern 0 (no loss) vs. patterns 3 and 5 (probable CCCA).
Rucker et al.,^26^ 2011.	Cross-sectional (questionnaire)	n = 201 (F:201, M:0)	Chemical relaxers	Hair straightening was a risk factor for traction alopecia (OR = 2.2; (95%CI 1.1–4.5; p = 0.03). Straightening associated with cornrow braids: OR = 5.27 (95% CI 1.5–18.32; p = 0.009).
Ogunbiyi et al.,^27^ 2014.	Case report	n = 1 (F:1)	Chemical relaxers	Hair loss associated to generalized proximal trichorrhexis nodosa, with atopy as the baseline condition.
Amorim et al.,^32^ 2017.	Cross-sectional	n = 30 (F:30, M:0)	Sodium hydroxide or guanidine hydroxide.	Areas of permanent alopecia were found where an artificial hair implant of Italian-knot type had been associated with hair straightening.

n, Number of patients; F, Female; M, Male.

**Table 6:** Studies on hair straighteners with morphological and physiological assessments.

Author	Study type	Population	Hair straightener	Alterations in hair strand and scalp
Galliano et al.,^28^ 2010.	Clinical trial	n = 12 (6 Chinese hair samples and 6 Caucasian hair samples)	Thiol straightener	Increased inter-scale distance, scale inclination and edge irregularity.
				Average size of particles extracted from hair after stirring in water for 30 minutes: different between ethnicities and proportionally greater increase in Chinese hair.
Kaliyadan et al.,^29^ 2016.	Cross-sectional	n = 25 (F:25) (19 subjects and 6 controls)	Chemical relaxers	In straightening only (n = 2): curly hair had grade 2 damage (severe cuticle lift, fissures or gaps with exposed cortex) and wavy hair had grade 0 (intact cuticle).
				In straightening associated with hair dye (n = 7): damage degrees from 1 (irregular cuticle without fissures or gaps) to 2.
				In the association of straightening, discoloration and hair dye (n = 1): grade 3 damage (partially exposed cortex).
				In the control group (wavy or straight hair): damage degree from 0 to 1.
Bloch et al.,^30^ 2019.	Clinical trial	Samples of Caucasian hair (straight dark brown; straight blond; wavy dark brown; and curly dark brown) and Afro hair.	Ammonia thioglycolate	71% reduction in Work (in Joules) for the combing of Afro hair.
				Greater color variation in dark brown and Afro-ethnic hair.
				Decreased tensile strength in all hair types.
				No additional loss of protein or tryptophan (except in Afro hair).
				Afro, curly and wavy dark brown hair: discreet opening of the scales, fissures and breakage.
Lee et al.,^31^ 2014.	Clinical trial	Asian, Caucasian, European and African American hair samples.	Ammonia thioglycolate	In straightening: Asian hair cuticles were the most resistant. The damage to the cortex was similar in the three groups.
				In straightening combined with commercial dye: the African American cuticle and cortex were the most resistant.
				The three groups showed similar patterns of cell membrane damage after straightening or straightening combined with dye.
França-Stefoni et al.,^11^ 2015.	Clinical trial	Dark brown curly Caucasian hair.	Ammonia thioglycolate, guanidine hydroxide, sodium hydroxide	Additional protein loss in washing: dye (48%), ammonia thioglycolate (159%); guanidine hydroxide (188%); sodium hydroxide (276%).
				When combined with dye, ammonia thioglycolate and guanidine hydroxide did not cause a significant additional increase in protein loss. Sodium hydroxide did.
Dyer et al.,^33^ 2013.	Clinical trial	European straight hair samples	Sodium hydroxide	Increased oxidative alterations, alkali-associated damage, increased dehydroalanine formation and side chain dehydration, and cysteine damage.
McMullen et al.,^34^ 2011.	Clinical trial	Samples of dark brown European hair and Piedmont hair	Sodium hydroxide	Degradation of tryptophan and its metabolites.
Dos Santos et al.,^35^ 2019.	Clinical trial	Afro and Caucasian white hair samples	Ammonia thioglycolate	Fiber lesions, with irregularities and cuticle detachment, and small undulations along its extension.
				Association of straightening with discoloration and straightening iron: irregular-contour hairs, cuticle detachment and deformation, and possible cortical damage.
Mamabolo et al.,^36^ 2013.	Clinical trial	N: 5 (F:5, M:0)	Sodium hydroxide and guanidine hydroxide	Guanidine hydroxide: greater softness, smooth appearance, shine, and less dry appearance.
				The two products were similar regarding damage.
				The no-lye straightener showed fewer split ends.
				Cystine content is reduced with lye straightener and more intensely with no-lye straightener.
				Lysine decreased in relation to virgin hair for the two straighteners; however, with no statistical difference between them.
Khumalo et al.,^37^ 2010.	Historical cohort	n = 30	Chemical relaxers	Cystine, citrulline (not always with statistical significance) and arginine decreased and glutamine increased in straightened hair. Cystine levels were lower in treated hair than in virgin hair, with levels similar to those found in trichothiodystrophy.
Beach et al.,^38^ 2012.	Clinical trial	n = 36 (F:30, M:0)	Guanidine hydroxide, sodium hydroxide, ammonium thioglycolate	IL-1α and IL-1ra were detected on the scalp.
				After the straightening, the levels of IL-1α and IL-1ra on the crown and vertex were lower, but with no difference compared to the group that did not straighten the hair.
				It was not possible to demonstrate changes in specific inflammatory cytokines after use of straighteners.
Tackey et al.,^39^ 2013.	Clinical trial	Three-dimensional epidermis model	Sodium hydroxide, guanidine hydroxide	IL-1α: at 4h of application there was no significant difference between the groups of straighteners, and at 24h and 48h the no-lye straightener reached higher levels.
				IL-1ra: always higher with no-lye.
				Both types had similar IL-1ra/IL-1α ratios in the early phases(suggestive of lack of relationship with immediate discomfort after application) and late phases; however the no-lye straightener had a higher ratio at 24h.
				PGE2 levels were higher than in controls at all phases.

n, Number of patients; F, Female; M, Male.

**Table 7:** Studies about prevention of structural damage during the use of hair straighteners.

Author	Study type	Population	Hair straightener	Effects on hairs
De Sá Dias et al.,^40^ 2008.	Clinical trial	Afro hair samples	Ammonia thioglycolate	Conditioning agents added to the straightener resulted in less protein loss, in fiber protection and in increased strength to breakage. However, it may reduce the effectiveness of straightening.
Bernard et al.,^41^ 2002.	Clinical trial	African-American hair samples	Guanidine hydroxide or sodium hydroxide	The use of ceramides (in this case, C18-dhCer) prevented damage to straightened hair.
Vermeulen et al.,^42^ 2004.	Clinical trial	Hair samples	Sodium hydroxide, lithium hydroxide	Addition of polymethylene waxes to a straightening formula resulted in a better appearance compared to commercial formulas, without minimizing performance.

### Straighteners in general

Regarding exposure to straighteners, Marks et al. compared patients with cicatricial alopecia and patients with non-cicatricial alopecia (control group). There was a significant difference in the groups regarding prior history of chemical straightening (25% in the scarring alopecia group and 13% in the non-scarring group), mainly due to patients with lichen planopilaris (LPP) and frontal fibrosing alopecia (FFA).^43^ This study does not specify which straighteners were used by the subjects; however, it mentions sodium hydroxide and glutaraldehyde as examples.^43^ The summary of results is shown in Table 8.^43^

**Table 8:** Studies on the effects of acid straighteners and traditional straighteners (hair relaxers).

Author	Study type	Population	Straightener agent	Alterations
Marks et al.,^43^ 2019.	Case-control	n = 286 (n = 43 patients with cicatricial alopecia), (143 patients with non-cicatricial alopecia: control group)	Chemical straightener	Higher frequency of hair straightening in the cicatricial alopecia group (25%) compared to the control group (13%).
				Prior history of straightening in 21% in LPP/FFA group and in 10% of paired controls.
				CCCA: more cumulative exposure to straighteners.

n, Number of patients; F, Female; M, Male.

## Discussion

Straighteners based on formaldehyde or derivatives consist, in general, of aldehyde and proteins such as hydrolyzed keratin: after exposure to heat, the aldehyde would promote a cross-link between the keratins in the product and those in the hair.^9^ Supposedly, straightening occurs by diffusing keratin into the cortex, filling in the defects, and restructuring the hairs; however, there is no evidence of this effect.^9^ The use of formaldehyde and glutaraldehyde as hair straighteners is not legal in Brazil.^7^

Products with glyoxylic acid are mentioned on the ANVISA website as glyoxyloyl-carbocysteine ​​and glyoxyloyl keratin amino acids in the section “recommendations on straighteners - what should I find on the label of these products”, with the consideration that they must warn the consumer about the risk of hair loss or change in hair color.^7^ However, the substance does not appear in the NI n. 64 of the organization.^10^ Carbocisteine ​​is not a straightener when used alone, but it is usually combined with glyoxylic acid.^8^

Acid straighteners remain a constant in straightening products. Locally, formaldehyde can cause dermatitis, including allergic eczema and psoriasiform eczema.^12^, ^13^ The medical literature reports of scalp reactions may be underestimated, as irritant contact dermatitis following formaldehyde use is often considered to be a normal effect by users. In the hair shaft, formaldehyde and glyoxylic acid, despite the macroscopic appearance of hair shine, worsened cuticle irregularity on electron microscopy and decreased resistance to breakage.^14^ However, other studies have not shown the same damage.^15^, ^16^ Although there are changes in strength, hair color, and other evidence of damage to the shaft, the Work for the combing the hair decreases with acidic straighteners, as shown in a study with glyoxylic acid.^17^

As for the straighteners permitted by law in Brazil, they comprise those listed in NI n. 64 of ANVISA.^10^ The hydroxides act due to their high pH, ​​opening the cuticles and allowing the action of the alkaline reducing agent in the cortex, where the disulfide bonds are broken. The hair is then mechanically straightened with the restructuring of the bonds between the keratins. Then, the hair is exposed to the acidic substance to terminate the process and close the cuticles (neutralization process).^1^, ^8^, ^44^ Alkalis react with cystine and produce lanthionine, a process called lanthionization. This process replaces one-third of the cystine with lanthionine.^8^ There is no need for heat or external traction.^8^ In contrast, thioglycolate does not act by lanthionization, but through a selective reduction of cystine bonds without changing the entire protein, being neutralized with hydrogen peroxide or sodium bromate, and the bonds are rearranged.^8^, ^44^ Heat should be applied for the heat treatment step.^8^, ^44^ Its use is commonly referred to as the “Japanese brushing”.^8^ It maintains 90% of the cystine content, and 10% becomes cysteic acid.^8^ There is also ammonium bisulfite, which is less aggressive, less effective, and more commonly used in household products.^3^

Relaxer-type straighteners are not free of side effects. Allergies, hair shaft frizz, dry hair, thinning and weakening of hairs, trichoptilosis, and hair color change have been reported.^18^, ^19^, ^20^, ^30^ On the scalp, desquamation, pain, atrophy, even burns, and potentially severe cases of contact eczema with secondary infection have been reported.^18^, ^19^, ^20^, ^21^

The long-term consequences of acute inflammation are not known, but they are even thought to be related to central centrifugal cicatricial alopecia.^22^ One hypothesis would be that cytokine changes caused by straighteners might play a role in the inflammatory process.^38^ The role of cytokines, including their relationship to the sensory diferences among several types of products, has been studied.^39^

Hair loss has also been described , including irreversible alopecia.^18^, ^19^, ^20^ Data regarding the association with CCCA diverge.^23^, ^24^, ^25^, ^43^ There is an increased risk of traction alopecia with relaxers, especially if associated with cornrow braids, and generalized proximal trichorrhexis nodosa was associated with hair straightening.^26^, ^27^

The hair shaft, with the use of relaxers, show an increase in the inter-scale distance, the opening of the scales, irregularity of their edges, fissures, severe lifting of the cuticle with exposure of the cortex, damage to the fiber, increase in particles extracted from them.^28^, ^29^, ^30^, ^35^

The use of straighteners concomitantly with other hair techniques seems to increase the vulnerability to damage. Hair relaxing in addition to the use of artificial hair implants has been related to areas of permanent alopecia.^32^ Hair relaxers resulted in a greater propensity for protein loss, and when combined with hair dye, sodium hydroxide was even more aggressive.^11^ The summation of ammonium thioglycolate straightening, discoloration, and straightening iron resulted in hairs with irregular contours, cuticle detachment and deformation, and possible cortical damage.^35^

Regarding lye and no-lye straighteners, a difference was found in the number of amino acids of treated hair compared to virgin hair. The decrease in cystine, present in the disulfide bonds that give hair strength, is evident.^37^ Cysteine deterioration was observed at the molecular level in European straight hair exposed to sodium hydroxide^33^ and straightening with lye relaxer degraded tryptophan;^34^ however, the use of thioglycolate did not result in additional loss of protein or tryptophan.^30^ Comparing lye and no-lye straighteners, there was a reduction in cystine after the use of both, with a greater loss with no-lye straighteners.^36^

Hairs of different ethnicities seem to diverge regarding their susceptibility to straightening, with mixed findings in the literature.^31^, ^35^ Structural damage can be minimized by using conditioning agents, some ceramides, and waxes.^40^, ^41^, ^42^ Despite the side effects, many women choose to straightening their hair.^19^, ^25^

The study limitations were: the search was restricted to three databases only; the search was carried out in two languages only; there is a scarcity of research, resulting in low scientific rigor for article inclusion; accepting different study designs.

## Conclusion

The review of data related to the last 20 years, despite limitations, discloses the significant occurrence of alterations in the hair shaft and scalp secondary to hair straightening. Clinical and subclinical inflammation of the scalp, damage to hair, hair loss due to breakage were evident. The long-term effects are still unknown.

Health professionals, especially dermatologists, as well as beauty salon professionals and their clients, need to know about the risks and benefits of these chemical treatments.

ANVISA’s recent norm limits the registration of straighteners; however, it does not clarify the new “acid straighteners”, such as glyoxylic acid.^10^ The effects of acidic straighteners are a matter for research, especially because of the Brazilian multicenter study that hypothesized the association of frontal fibrosing alopecia with formalin-straightening procedures.^45^

Studies carried out with scientific rigor are necessary to better understand the responses of hair structures to chemical agents, especially their relationship with the development of alopecia.

This research did not receive any specific funding from public, private or nonprofit funding agencies.

## Financial support

None declared.

## Authors’ contributions

Joane Nathache Hatsbach de Paula: Design and planning of the study; collection, analysis, and interpretation of data; critical review of the literature; drafting and editing of the manuscript.

Flávia Machado Alves Basílio: Design and planning of the study; effective participation in research orientation; critical review of the manuscript; approval of the final version of the manuscript.

Fabiane Andrade Mulinari-Brenner: Effective participation in research orientation; critical review of the manuscript; approval of the final version of the manuscript.

## Conflicts of interest

None declared.
